# An Analysis of the Effects of Vancomycin and/or Vancomycin-Resistant *Citrobacter freundii* Exposure on the Microbial Community Structure in Soil

**DOI:** 10.3389/fmicb.2016.01015

**Published:** 2016-06-28

**Authors:** Mariusz Cycoń, Sławomir Borymski, Kamila Orlewska, Tomasz J. Wąsik, Zofia Piotrowska-Seget

**Affiliations:** ^1^Department of Microbiology and Virology, School of Pharmacy with the Division of Laboratory Medicine, Medical University of SilesiaSosnowiec, Poland; ^2^Department of Microbiology, University of SilesiaKatowice, Poland

**Keywords:** vancomycin, antibiotic resistance, *Citrobacter freundii*, DGGE, PLFA, soil

## Abstract

The occurrence of antibiotics and antibiotic resistance genes in the environment has become a subject of growing concern. The extensive use of vancomycin and other pharmaceuticals may alter the biodiversity of soil microbial communities and select antibiotic-resistant bacteria. Therefore, the purpose of the study was to evaluate the impact of vancomycin and/or vancomycin-resistant *Citrobacter freundii* on soil microbial communities using the denaturing gradient gel electrophoresis (DGGE) and the phospholipid fatty acid (PLFA) approaches. The experiment had a completely randomized block design with the following treatments: control soil (C), soil with vancomycin (1 mg/kg soil—VA1), soil with vancomycin (10 mg/kg soil—VA10), soil with *C. freundii* (Cit), soil with vancomycin (1 mg/kg soil) and *C. freundii* (VA1+Cit), and soil with vancomycin (10 mg/kg soil) and *C. freundii* (VA10+Cit). A bacterial strain resistant to vancomycin was isolated from raw sewage collected from the municipal sewage treatment plant. The obtained results indicated that the antibiotic and/or the bacterial strain exerted a selective pressure that resulted in qualitative and quantitative changes in the population of soil microorganisms. However, a multivariate analysis showed that the genetic and structural diversity of the soil microbial community was primarily affected by the incubation time and to a lesser extent by the antibiotic and introduced bacteria. DGGE analysis clearly showed that certain species within the bacterial community were sensitive to vancomycin as was evidenced by a decrease in the values of *S* (richness) and *H* (Shannon-Wiener) indices. Moreover, a PLFA method-based analysis revealed alterations in the structure of the soil microbial community as indicated by changes in the biomass of the PLFA biomarkers specific for Gram-positive and Gram-negative bacteria as well as fungi. The changes observed in the community of soil microorganisms may decrease the rate of microbial-mediated processes, which can lead to a disturbance in the ecological balance of the soil ecosystem.

## Introduction

The intensive use of antibiotics, their disposal and consequent presence in water and soils are of great concern regarding their ecotoxicological effect on the organisms of different trophic levels and the spreading of antibiotic resistance genes (ARGs; Kümmerer, 2009). Antibiotics are commonly used in human and veterinary medicine, livestock industries and agriculture. Such widespread use results in an increased concentration of antibiotics in water, sediments, and soils. Antibiotics enter the environment in various ways. The primary sources of pharmaceutical contamination include domestic, industrial, and hospital wastewater, as well as effluents from wastewater treatment plants (WWTPs), aquaculture and livestock farming (Bottoni et al., 2010). Since conventional WWTPs have a limited efficiency in removing antibiotics from wastewater, these chemicals are released into water bodies and soils (Li and Zhang, 2010). Moreover, the application of manure and sewage sludge as fertilizers can contribute to the spread of pharmaceuticals and antibiotic-resistant microorganisms into soil (Kümmerer, 2003; Bondarczuk et al., 2016). For example, Su et al. (2015) found 156 ARGs encoding resistance to various antibiotics in composted sewage sludge showing that this by-product is a reservoir of antibiotic-resistance determinants.

The amount of antibiotics in water ranges from nanograms per liter in surface water to micrograms and milligrams per liter in effluents from hospital, an antibiotic drug manufacturers and WWTPs (Brown et al., 2006; Larsson et al., 2007; Tamtam et al., 2011). The results of many studies have provided evidence of the presence of many antibiotics in the soil at concentrations ranging from ng/kg soil to hundreds of μg/kg soil (Aust et al., 2008; Karci and Balcioglu, 2009). Although the concentration of environmental antibiotics is not high, recent studies have shown that even low concentrations of antimicrobials select antibiotic-resistant bacteria, maintain the ARGs and favor horizontal gene transfer (Kim et al., 2003; Gullberg et al., 2011, 2014). Xiong et al. (2015) observed that the tetracycline present in manure polluted freshwater-sediment was important for maintenance of tet genes encoding resistance of bacteria to tetracycline. Apart from the emergence of antibiotic-resistant bacteria, antibiotics present in soil may negatively affect the activity and biodiversity of microbial communities. It has been reported that antibiotics alter the number of bacterial and fungal cells, the rate of soil respiration, carbon mineralization, and nitrogen cycling (Čermák et al., 2008; Conkle and White, 2012; Rosendahl et al., 2012; Banerjee and D'Angelo, 2013; Cui et al., 2013). A key factor that determines the adverse impact of antibiotics on soil microorganisms is their persistence. The half-life of various antibiotics in soils, which depends both on the antibiotic properties and soil characteristics, varied from a few days to as many as 300 days (Hektoen et al., 1995; Weerasinghe and Towner, 1997; Ingerslev and Halling-Sorensen, 2001; Gartiser et al., 2007). The important antibiotic properties include their molecular structure, photo stability, water solubility, biodegradability, and their ability to bind to the soil matrix (Sukul et al., 2008; Förster et al., 2009; Figueroa-Diva et al., 2010; Hu et al., 2010; Fatta-Kassinos et al., 2011; Kwon, 2011).

One of the antibiotics that has been used for more than 60 years is vancomycin. It is a bactericidal glycopeptide used to manage severe Gram-positive infections including septicemia, bone infections, lower respiratory tract infections, skin and skin structure infections. Vancomycin exhibits its antibacterial activity by inhibiting the later stages of cell wall synthesis, and thus affects dividing bacteria (Gupta et al., 2011). When vancomycin binds to the D-alanyl-D-alanine residue of pentapeptide, it blocks the addition of late precursors to the peptidoglycan (PG) chain and inhibits the subsequent cross-linking of PG (Courvalin, 2006). However, in recent years bacteria with the reduced susceptibility to vancomycin has been isolated in many countries all over the world (Sujatha and Praharaj, 2012). Moreover, the first resistant strain of vancomycin-resistant *S. aureus* was reported in 2002 (Chang et al., 2003). The basic mechanisms of vancomycin resistance involve the synthesis of precursors with low-affinity to antibiotics and the removal of the vancomycin-binding target through the elimination of the high-affinity precursors (Arthur et al., 1996). Vancomycin has been detected at concentrations reaching a concentration of 37.3 μg/L in hospital effluents (Passerat et al., 2010) and at a concentration of 24 ng/L in waste water effluents (Zuccato et al., 2010). During the activated sludge process in WWTPs, only 52% of the vancomycin is eliminated (Li and Zhang, 2011), and therefore, it has been found at concentrations ranging from 0.44 to 5.17 ng/L in surface water. Moreover, several reports have documented the presence of vancomycin-resistant bacteria in municipal WWTPs, effluents from hospital and surface water (Nagulapally et al., 2009; Łuczkiewicz et al., 2010; Morris et al., 2012).

Soil microbial communities play a critical role in the proper functioning of the environment and the characterization of these communities exposed to antibiotics and/or antibiotic-resistant bacteria will provide valuable information for the sustainable management and quality of soil. The presence and accumulation of vancomycin and other antibiotics in soil may have a deleterious effect on microbial communities and cause long-lasting changes. There is still little information related to the effect of vancomycin and vancomycin-resistant bacteria on the total microbial community structure in soil. Therefore, the objective of the present study was to determine the structural and genetic diversity of a soil microbial community as determined by the phospholipid fatty acid (PLFA) and the denaturing gradient gel electrophoresis (DGGE) methods in vancomycin and/or vancomycin-resistant bacteria-treated soil.

## Materials and methods

### Isolation of vancomycin-resistant bacteria

Raw sewage collected from the municipal sewage treatment plant “Gigablok” located in Katowice-Szopienice, southern Poland was the source of the bacterial strains that are resistant to vancomycin. The isolation procedure was performed using a TSA (Tryptone-Soya Agar) medium (BTL, Poland) and paper discs impregnated with 30 μg vancomycin (VA) (Oxoid, UK). The inoculated plates were incubated for 48 h at 30 ± 1°C. In order to obtain a pure culture of vancomycin-resistant bacteria, colonies located directly on the shore disc were transferred onto a new TSA medium and incubated under the same conditions. The individual bacterial colonies were selected and subcultured to obtain pure culture based on their morphological properties. One bacterial isolate was used for further analyses.

### Identification of bacteria

The isolate was characterized and identified using a biochemical test and 16S rRNA gene analysis as it was previously described by Cycoń et al. (2014). The biochemical properties of the isolate and the substrate utilization pattern were determined using an API 20E System (bioMérieux Inc., France) according to the manufacturer's recommendations. For the 16S rRNA sequence analysis, the genomic DNA was extracted from a strain collected at the late exponential stage of growth using a GeneMATRIX Bacterial and Yeast Genomic DNA Purification Kit (Eurx, Poland) as described in the protocol of the manufacturer. The 16S rRNA gene of the isolate was amplified using the universal primer pair: 27f (5′-AGA GTT TGA TCC TGG CTC AG-3′) and1492r (5′-TAC GGT TAC CTT GTT ACG ACT T-3′; Lane, 1991) obtained from Sigma-Aldrich (Germany). Amplification was performed using a PCR Master Mix Kit (Promega) according to the manufacturer's recommendations and a PTC-118 Thermal Cycler (Bio-Rad, CA, USA) under the following conditions: (i) an initial denaturation step of 95°C for 2 min, (ii) 30 cycles of denaturation, annealing, and extension (95°C for 1 min followed by 54°C for 30 s, with an extension step at 72°C for 2 min), and (iii) a final extension at 72°C for 5 min. After amplification the products were purified with a GeneMATRIX PCR/DNA Clean-Up Purification Kit (Eurx, Poland) according to the protocol of the manufacturer before the amplicons were sequenced. Gene sequencing was performed using a Big Dye® Terminator Cycle Sequencing Kit (Applied Biosystem) and an AbiPrism®3100 Genetic Analyzer. The obtained sequence was compared to known 16S rRNA gene sequences using the BLAST server at the National Center for Biotechnology Information (NCBI; http://www.ncbi.nlm.nih.gov/). DNA sequences were aligned using CLUSTAL W. Phylogenetic analysis was performed by the neighbor-joining (NJ) method, which tests the support for the phylogeny with a bootstrap analysis based on 1000 replicates using the MEGA ver. 6.0 software.

### Determination of the susceptibility of bacteria to selected antibiotics

The sensitivity assays to vancomycin and other selected antibiotics for isolated bacterial strain were performed on a Mueller-Hinton agar (BTL, Poland) using the Kirby–Bauer disc diffusion and the *E*-test methods according to the protocols for determining the zones of growth inhibition and the values of the minimum inhibitory concentrations (MICs), respectively. In order to prepare the inoculum, the bacterial strain were cultured on nutrient agar (BTL, Poland) for 24 h at 35 ± 1°C, and at the exponential phase, the bacteria were suspended in 0.85% sterile NaCl to obtain the bacterial suspension at a concentration of ~1.5 × 10^8^ cells/mL. The cell density (OD 550 nm) was measured using a densitometer (Densimat®, bioMérieux, France). Next, the bacterial suspension was inoculated on a Mueller-Hinton agar using a sterile cotton swab. In the case of the disc diffusion method, the paper discs impregnated with ciprofloxacin (CI: 5 μg), clindamycin (CM: 2 μg), erythromycin (EM: 15 μg), streptomycin (SM: 300 μg), tetracycline (TC: 30 μg), vancomycin (VA: 30 μg), or cefuroxime (XM: 30 μg) obtained from Oxoid (UK), were applied to the surface of an agar plate inoculated with the test strain. The inoculated plates were incubated for 24 h at 35 ± 1°C, after which, the diameters of the zones of growth inhibition around each disc were measured. In the case of the *E*-test method, strips with a defined gradient of ciprofloxacin (CI: 0.002–32 μg/mL), clindamycin (CM: 0.016–256 μg/mL), erythromycin (EM: 0.016–256 μg/mL), streptomycin (SM: 0.064–1024 μg/mL), tetracycline (TC: 0.016–256 μg/mL), vancomycin (VA: 0.016–256 μg/mL), or cefuroxime (XM: 0.016–256 μg/mL) obtained from bioMérieux (France) were applied to the surface of the agar plate inoculated with the test strain. The inoculated plates were incubated for 48 h at 35 ± 1°C. The MIC values were read from the scale in terms of μg/mL at the site where the ellipse edge intersected with the strip.

### Soil characteristics

Sandy loam soil samples were collected from the top layer (0–20 cm) from grass-covered fields located in the vicinity of Żywiec, southern Poland. The detailed physico-chemical properties of the soil (Table 1) were determined according to ISO standards as presented in previous paper (Cycoń et al., 2010). In the laboratory, the soil was sieved to a maximum particle size of <2 mm and immediately used for the experiment.

**Table 1:** **General characteristic of the soil used in the experiment**.

**Parameter**	**Value**
Sand (2000–50 μm) (%)	67.0 ± 4.2
Silt (<50–2 μm) (%)	24.0 ± 1.1
Clay (<2 μm) (%)	9.0 ± 1.3
Density g/cm^3^	1.35 ± 0.08
pH(in water)(1:5)	6.9 ± 0.3
Cation exchange capacity (CEC) (cmol+/kg)	10.0 ± 0.5
Water holding capacity (WHC)(%)	43.0 ± 1.2
Corg (%)	1.6 ± 0.2
Ntot(%)	0.15 ± 0.02
Microbial biomass (mg/kg)	932 ± 21

*The values are the means of three replicates with the standard deviation, which was within 5% of the mean*.

### Experimental design and treatments

**Figure 1:**
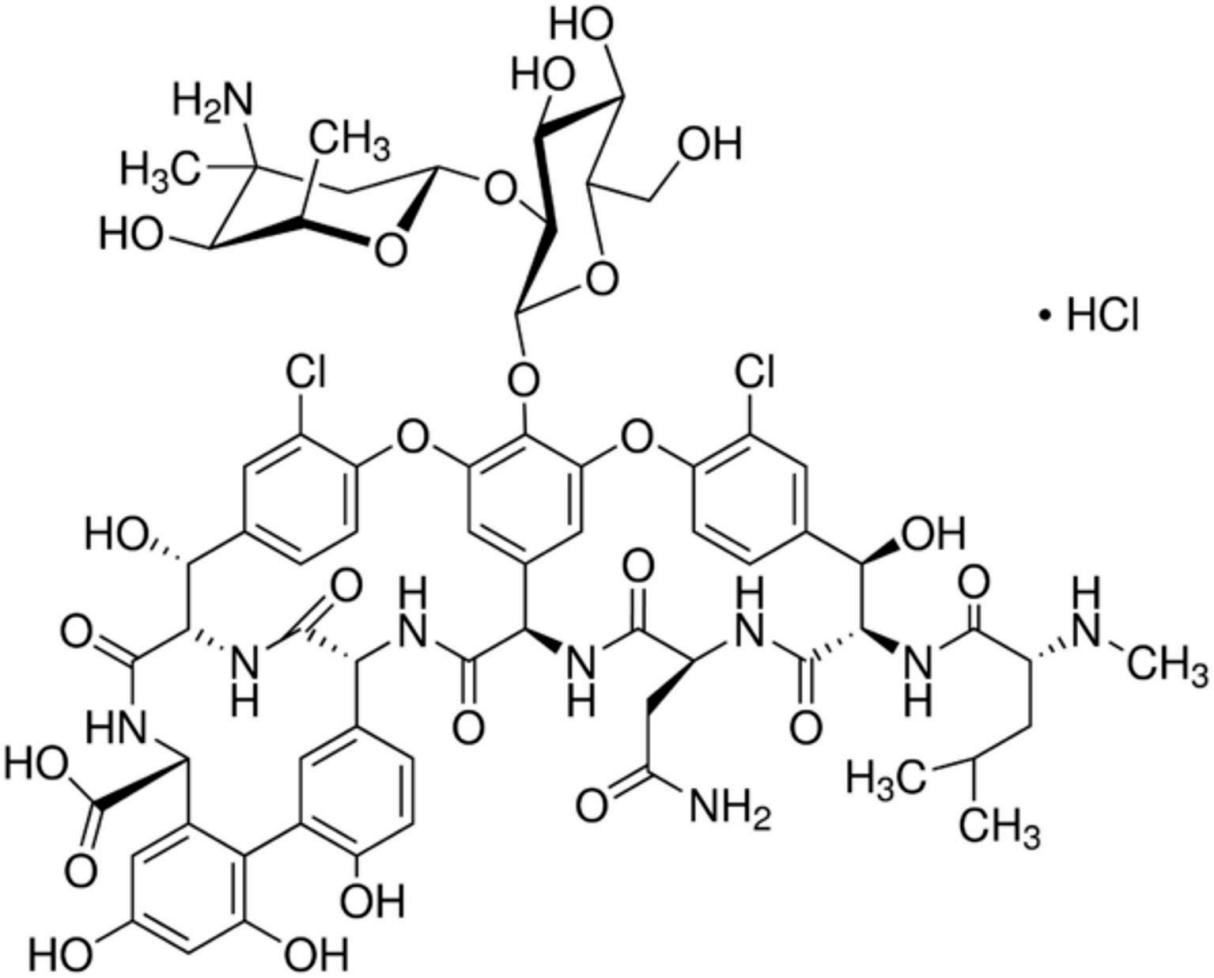
**Chemical structure of vancomycin**.

The European Pharmacopeia (EP) reference standard of vancomycin (C_66_H_75_Cl_2_N_9_O_24._ HCl; Figure 1) purchased from Sigma-Aldrich (Germany) was used in this study. The experiment had a completely randomized block design with the following treatments: control soil (C), soil with vancomycin (1 mg/kg soil—VA1), soil with vancomycin (10 mg/kg soil—VA10), soil inoculated with *Citrobacter freundii* (1.6 × 10^7^ cells/g soil; Cit), soil with vancomycin (1 mg/kg soil) and with *C. freundii* (1.6 × 10^7^ cells/g soil; VA1+Cit), and soil with vancomycin (10 mg/kg soil) and *C. freundii* (1.6 × 10^7^ cells/g soil; VA10+Cit). There were three replications of each treatment for each sampling time, which produced a total of 90 pots in the experiment (i.e., six treatments × three replications × five sampling times). In order to ensure an even distribution of the antibiotic in the soil, the VA solution was prepared in sterile pure water and then added to sterile quartz sand (<0.5 mm). After evaporation of water in the dark, the mixture of sand (50 g/kg soil) and VA was added into the soil portion and thoroughly mixed. The concentrations of vancomycin that were used reflect the most adverse scenarios associated with the entry of large quantities of antibiotics into the soil as a result of the uncontrolled disposal of unused drugs into municipal waste or depositing them in landfills.

In order to prepare the inoculum, the bacterial strain was cultured in 200 mL Erlenmeyer flasks containing 100 mL of nutrient broth (BTL, Poland). At the exponential phase, the bacteria were pelleted by centrifugation (5 min, 10,000 g). The pellet was washed twice with 0.85% of sterile NaCl and then resuspended in 0.85% of sterile NaCl to obtain a bacterial suspension at a concentration of ~2.1 × 10^9^ cells/mL. The cell density (OD 550 nm) was measured using a densitometer (Densimat®, bioMérieux, France). Next, the bacterial suspension was introduced into the soil in triplicate in order to produce a final bacterial count of ~1.6 × 10^7^ cells/g soil.

The water content of the soils was adjusted to 50% of the maximum water holding capacity and maintained at this level during the experimental period. The pots were covered with perforated polypropylene sheets and were incubated in the dark at 22 ± 1°C for 90 days. Soil samples were periodically taken (on days 1, 15, 30, 60, and 90) for the determination of the genetic and structural diversity of the microbial communities.

### Analysis of the soil microbial community structure using the PCR-DGGE method

Total DNA was extracted from the soil samples using a GeneMATRIX Soil DNA Purification Kit (Eur_x_, Poland) as described in the protocol of the manufacturer and subjected to electrophoresis in 1% (w/v) agarose gel followed by quantification using a Biophotometer (Eppendorf, Germany). A fragment of the V3 region of the bacterial 16S RNA gene was amplified using the primers F338 (5′-ACT CCT ACG GGA GGC AGC AG-3′) and R518 (5′-ATT ACC GCG GCT GCT GG-3′). The forward primers contained a 40-bp GC-clamp (5′-CGC CCG CCG CGC GCG GCG GGC GGG GCG GGG GCA CGG GGG G-3′) attached to the 5′ end (Muyzer et al., 1993). The PCR reaction mixture contained 1 × GoTaq Flexi Buffer (Promega), 2 mM MgCl2, 0.2 mM dNTP Mix (Promega), 0.5 μM of each primer (Sigma-Aldrich), 0.2 μg of DNA, and 1.5 U/μL GoTaq DNA Polymerase (Promega). PCR was carried out using a PTC-118 Thermal Cycler (BIO-RAD, CA, USA) as follows: (i) an initial denaturation step of 95°C for 10 min, (ii) 30 cycles of denaturation, annealing, and extension (95°C for 1 min followed by 53°C for 1 min with an extension step at 72°C for 2 min), and (iii) a final extension at 72°C for 12 min.

After amplification the products were purified using a QIAquick PCR Purification Kit (Qiagen, USA) according to the protocol of the manufacturer and then analyzed in 8% (w/v) polyacrylamide gel (37.5:1 acrylamide:bis-acrylamide), composed of a linear denaturing gradient ranging from 40 to 70%. Denaturant solutions were prepared by mixing the appropriate volumes of two (0–100%) denaturant stock solutions (7 mol/L urea and 40% v/v formamide). The electrophoresis was run at 60°C in a 1 × TAE buffer for 14 h at a constant voltage of 80 V using a DCode Mutation Detection System (Bio-Rad, USA). After this, the gels were stained with ethidium bromide (0.5 mg/mL) and visualized on a UV trans illuminator.

The DGGE profiles were analyzed using BioNumerics software ver. 7.5 (Applied Math, Belgium). The similarity values of each DGGE band was calculated by applying the Dice coefficient. Based on the presence/absence of a band and band weighting (band density) analyses, phylogenic dendrograms were constructed using the unweighted pair-group method and the arithmetic averages (UPGMA). Richness (*S*) values were calculated as the number of DNA bands detected in the respective lines of the DGGE profile, while the Shannon–Wiener index (*H*), and evenness (*E*_*H*_) values were calculated according to the Equations (1) and (2), respectively.

(1) $$\begin{array}{r} H=-\Sigma pi\left(\ln\;pi\right) \end{array}$$

(2) $$\begin{array}{r} E_{H}=\frac{H}{H_{\max}}=\frac{H}{\ln\;S} \end{array}$$

where *p*_*i*_ is the ratio between the specific band intensity and the total intensity of all of the bands and *S* is the total number of bands in each sample.

### Analysis of the soil microbial community structure using the PLFA method

The community structure of the total microbial community was assessed using a slightly modified protocol for a PLFA analysis by Frostegård et al. (1993). Briefly, the lipids from 2 g of soil were extracted using a solution containing a chloroform:methanol:citric buffer (1:2:0.8 v/v/v) and separated into neutral, glycolipid and phospholipid fractions in silicic acid columns (Supelco Silica Tube, 3 mL, 500 mg). In order to obtain the fatty acid methyl esters (FAMEs), the phospholipids were subjected to a mild alkaline methanolysis. FAMEs were subsequently analyzed using an Agilent 7820A GC gas chromatograph system with an Agilent HP-Ultra 2 capillary column (cross-linked 5% phenyl-methyl silicone; 25 m, 0.20 mm ID; film thickness 0.33 μm) with hydrogen as the carrier gas. FAMEs were detected using a flame ionization detector (FID) and identified using the MIDI Microbial Identification System software (Sherlock TSBA6 library; MIDI Inc., Newark, DE, USA). Nonadecanoic acid (19:0) was used as the internal standard for the fatty acid quantitative analysis. Total PLFA biomass was calculated by summing all of the isolated fatty acids. Bacterial biomass was calculated based on fatty acids of a bacterial origin. The community structure analysis was based on the fatty acids that are thought to be the biomarkers of Gram-positive bacteria (i15:0, a15:0, i16:0, i17:0, a17:0) and Gram-negative bacteria (16:1ω7t, 18:1ω7, cy19:0, cy17:0). The distribution of 18:2ω6 as well as 10Me 17:0 and 10Me 18:0, was used to determine fungal biomass and actinomycetales, respectively (Frostegård and Bååth, 1996; Bååth and Anderson, 2003). Additionally, the ratios of the biomass of Gram-positive to the biomass of Gram-negative bacteria as well as the bacterial to fungal fraction were calculated.

### Statistical analyses

The DGGE and PLFA data were analyzed using a three-way analysis of variance (ANOVA), which allowed the percentage of the variation attributable to the antibiotic concentration, bacterial strain, and incubation time to be determined. The statistical significance of differences (*P* < 0.05) in the measured data was assessed by a *post-hoc* comparison of the means using the least significant differences (LSD) test. A principal component analysis (PCA) of the microbial PLFA biomarkers of Gram-positive and Gram-negative bacteria as well as fungi was carried out in two sets that included the PLFA data from all of the sampling days and were performed separately for each sampling day. The three-way and two-way MANOVA analyses of the PC scores were performed for the first and second PCA sets, respectively. The PCA analyses were performed on the standardized data. All statistical analyses were performed using the Statistica 12.0 PL software package.

## Results

### Isolation and identification of vancomycin-resistant bacteria

**Figure 2:**
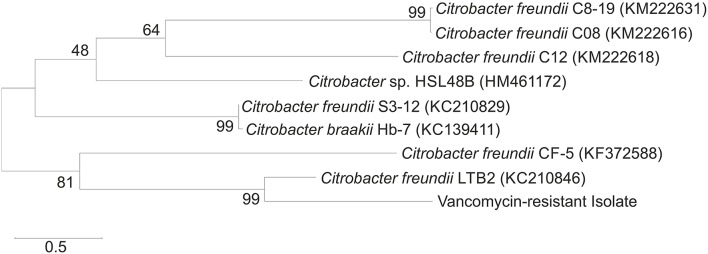
**Neighbor-joining phylogenetic tree of the bacteria based on comparisons of the 16S rRNA gene sequences**. Bootstrap values from 1000 replications are indicated at the branches. GenBank accession numbers are given in parentheses.

The isolation procedure enabled isolation of the bacterial strain that was characterized by the tolerance to vancomycin applied at a concentration of 30 μg/mL. After incubation on nutrient agar plates (24 h), the colonies of the isolated strain were observed to be creamy white. Identification based on the analysis of the partial 16S rRNA sequence allowed the isolated strain to be identified as *C. freundii*. The phylogenetic analysis showed that the 16S rRNA sequence of the isolate had a 99% sequence similarity with *C. freundii* LTB2 strain (Figure 2). Analysis of the biochemical pattern using an API 20 *E*-test system (Table 2) allowed the numerical profile (3,604,772) to be obtained, which was compared with profiles deposited in the apiweb™ database, and that also supported the reliable identification of the isolate as *C. freundii* with a 97.9% identity.

**Table 2:** **Biochemical pattern of bacterial strain isolated from raw sewage**.

**Characteristics**	**Results**	**Characteristics**	**Results**	**Characteristics**	**Results**
2-Nitrophenyl-β-D-galactopyranoside	+	L-Tryptophane (deaminase)	−	D-Sorbitol	+
L-Arginine	+	L-TRYPTOPHANE (indol)	−	L-Rhamnose	+
L-Lysine	−	Sodium pyruvate	−	D-Sacharose (sucrose)	+
L-Ornithine	−	Gelatin	−	D-Melibiose	+
Trisodium citrate	+	D-Glucose	+	Amygdalin	−
Sodium thiosulfate	+	D-Mannitol	+	L-Arabinose	+
Urea	−	Inositol	+	Oxidase	−

*+, tested positive; −, tested negative*.

### Susceptibility of *Citrobacter freundii* to selected antibiotics

The results obtained using the disc diffusion method showed that the isolated strain was resistant to clindamycin, erythromycin, and vancomycin as indicated by the lack of inhibition zones of bacterial growth around the discs impregnated with antibiotics at concentrations of 2, 15, and 30 μg/ml, respectively (Table 3). In general, the antibiotics for which there were no zones of the inhibition of the growth of the bacterial strain using the disc diffusion method showed no growth inhibition using the *E*-test, and their MIC values were higher than 256 μg/mL. On the other hand, the highest sensitivity of a bacterial strain to ciprofloxacin was determined for which the MIC value was 0.023 μg/mL (Table 3).

**Table 3:** **Results of susceptibility of isolated ***Citrobacter freundii*** to selected antibiotics**.

**Antibiotic**	**Disc-diffusion method**		***E*****-test method**	
	**Concentration (μg)**	**Zone of growth inhibition (mm)**	**Used range of concentrations (μg/mL)**	**MIC (μg/mL)**
CI	5	32	0.002−32	0.023
CM	2	−	0.016−256	>256
EM	15	−	0.016−256	>256
SM	300	48	0.064−1024	2
TC	30	19	0.016−256	12
VA	30	−	0.016−256	>256
XM	30	27	0.016−256	1

*CI, ciprofloxacin; CM, clindamycin; EM, erythromycin; SM, streptomycin; TC, tetracycline; VA, vancomycin; XM, cefuroxime, −, no inhibition zone. Values of the inhibition zones and MICs (minimum inhibitory concentrations) were determined after 24 and 48 h of incubation, respectively*.

### DGGE analysis

**Figure 3:**
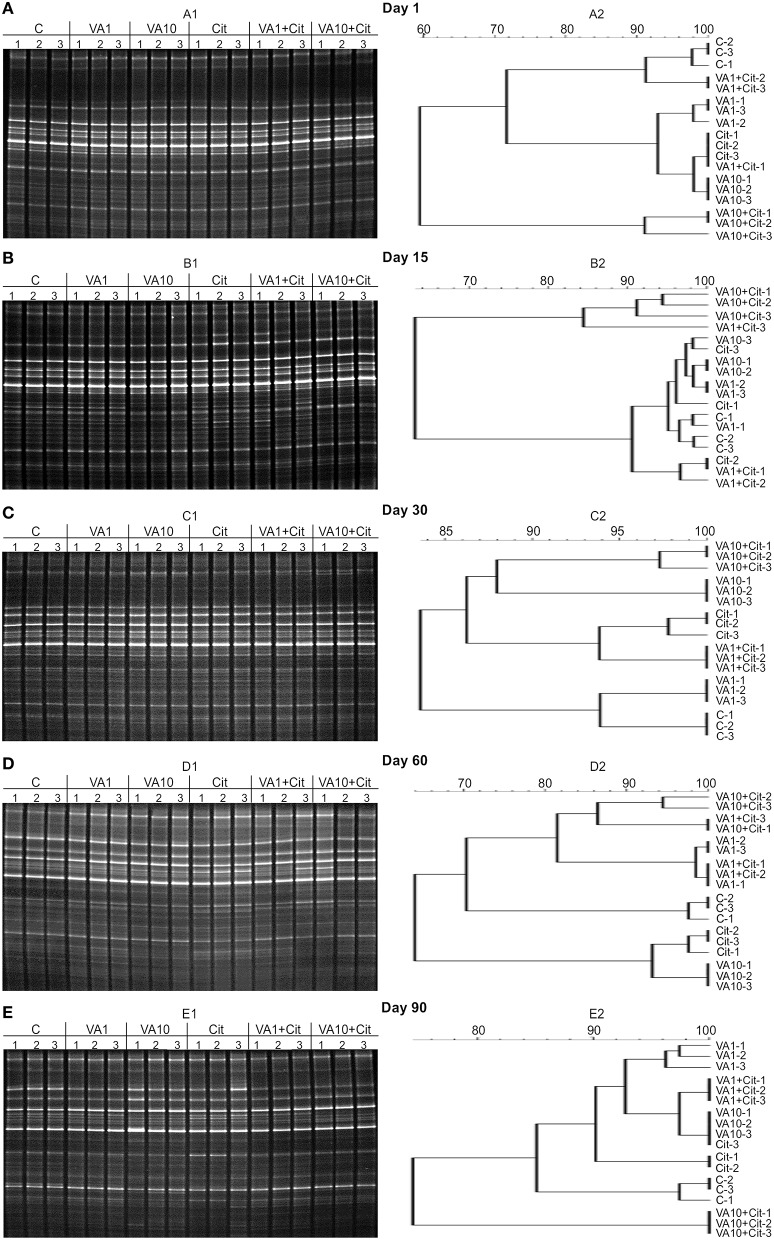
**DGGE profiles (A1–E1) and phylogenic dendrograms (A2–E2) for PCR-amplified fragments of the 16S rRNA gene for soil treated with vancomycin (VA) and/or ***Citrobacter freundii*** (Cit) on days 1 (A), 15 (B), 30 (C), 60 (D), and 90 (E) following treatment (C, control; VA1, 1 mg/kg soil; VA10, 10 mg/kg soil)**.

Analysis of the DGGE patterns revealed that vancomycin and/or *C*. *freundii* affected the structure of the soil microbial community during the 90-day experiment. The DGGE profiles generated from the replicates for all of the treatments were very similar regarding complexity and band position. Cluster analysis generally showed that the vancomycin dosage was the main factor responsible for the separation of the profiles. The presence/absence of a DGGE band and band weighting (band density) analysis revealed that several bacterial community members were affected by the antibiotic treatment (Figure 3).

**Figure 4:**
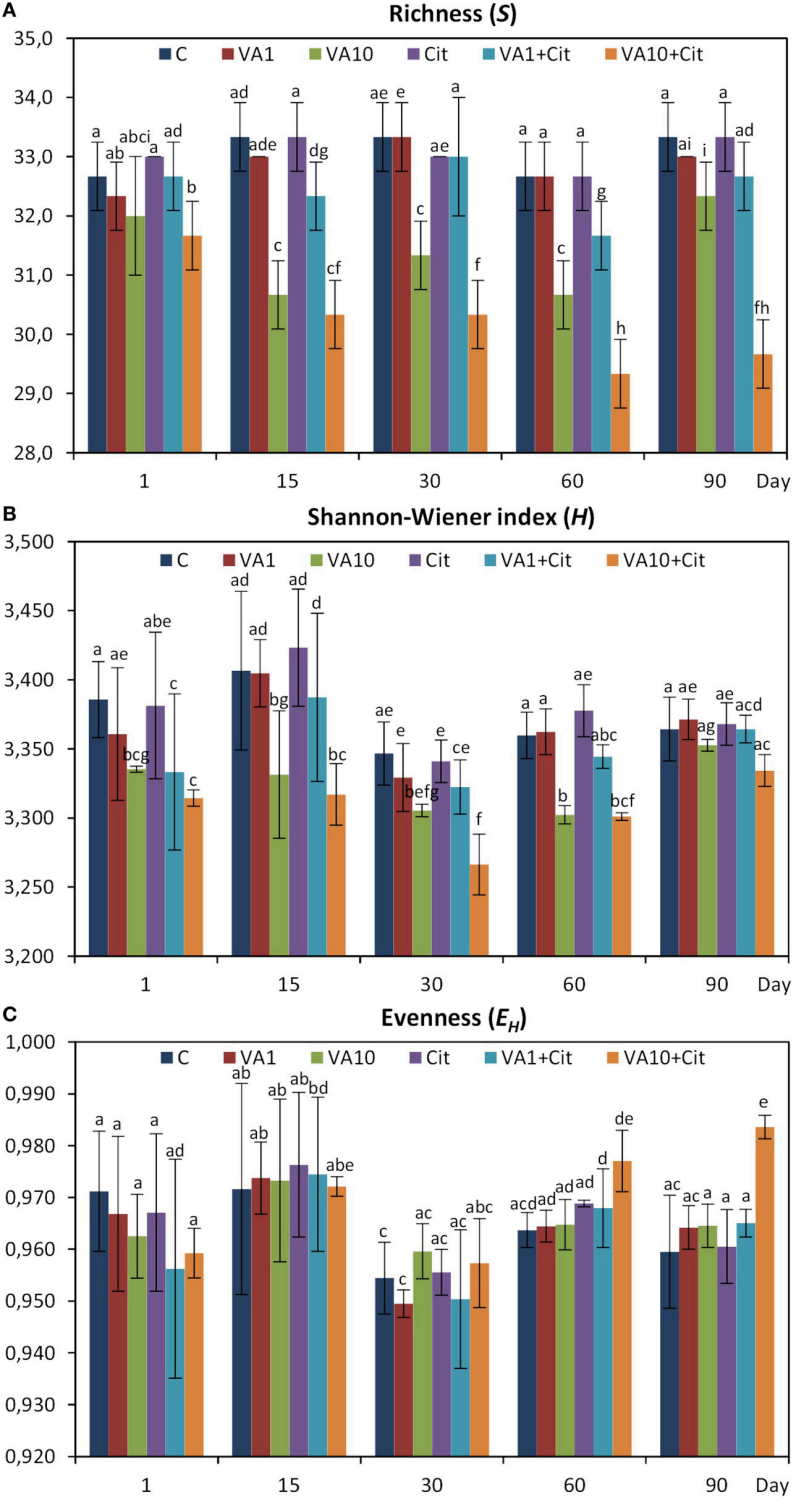
**Results of the DGGE analysis—***H***, Shannon–Wiener index (A); ***R***, richness (B); and ***E***_***H***_, evenness (C) for the control soil (C), soil with vancomycin (1 mg/kg soil)(VA1), soil with vancomycin (10 mg/kg soil)(VA10), soil with ***Citrobacter freundii*** (Cit), soil with vancomycin (1 mg/kg soil) and ***Citrobacter freundii*** (VA1+Cit) and soil with vancomycin (10 mg/kg soil) and ***Citrobacter freundii*** (VA10+Cit)**. The data presented are the means and standard deviations of three replicates. Different letters (within each index) indicate significant differences (*P* < 0.05, LSD test) related to the effects of the antibiotic dosage, bacterial strain, and time.

The calculation of the richness (*S*) and the Shannon–Wiener index (*H*) values indicated that especially the soil treated with the higher dosage of vancomycin and/or inoculated with *C*. *freundii* experienced significant (*P* < 0.05) changes in the overall richness and diversity of the dominant bacteria in comparison to the control soil during the experimental period (Figure 4). The three-way ANOVA analysis showed that the richness value was significantly affected by the bacterial strain (*P* < 0.001), the vancomycin dosage (*P* < 0.001), and the incubation time (*P* < 0.001; Table 4). The vancomycin dosage explained most of the variance (61%), whereas the effect of the bacterial strain accounted for 4% of the variance and the effect of time explained a further 6%. The interactions between the strain and vancomycin concentration (S × C) as well as the concentration and incubation time (C × T) were also significant and explained 4 and 6% of the variance, respectively (Table 4). In contrast, the *H* index was significantly affected only by the vancomycin dosage (*P* < 0.001) and the incubation time (*P* < 0.001), which explained 35 and 22% of the variance, respectively. The ANOVA also indicated that the incubation time was the only factor that significantly (*P* < 0.001) affected the *E*_*H*_ value during the experimental period and explained 30% of the variance (Table 4).

**Table 4:** **Results of three-way ANOVA for the effects of concentration of vancomycin, bacterial strain, time, and their interaction on DGGE parameters**.

**Parameter**	**Source of variation**	***df***	**Sum of squares**	**Mean squares**	**Variance explained (%)**	***F***	***P***
Richness (*S*)	Strain (S)	1	5.88	5.88	4	17.6	***P* < 0.001**
	Concentration (C)	2	85.09	42.54	61	127.6	***P* < 0.001**
	Time (T)	4	8.18	2.04	6	6.1	***P* < 0.001**
	S × C	2	4.96	2.48	4	7.4	***P* = 0.001**
	S × T	4	3.29	0.82	2	2.5	*P* = 0.054
	C × C	8	8.36	1.04	6	3.1	***P* = 0.005**
	S × C × T	8	4.04	0.51	3	1.5	*P* = 0.171
Shannon–Wiener index (*H*)	Strain (S)	1	0.002	0.002	1	2.4	*P* = 0.129
	Concentration (C)	2	0.056	0.028	35	32.7	***P* < 0.001**
	Time (T)	4	0.035	0.009	22	10.3	***P* < 0.001**
	S × C	2	0.003	0.001	2	1.5	*P* = 0.224
	S × T	4	0.001	0.0003	1	0.3	*P* = 0.874
	C × C	8	0.010	0.001	6	1.4	*P* = 0.211
	S × C × T	8	0.001	0.000	1	0.2	*P* = 0.992
Evenness (*E_H_*)	Strain (S)	1	0.00008	0.00008	1	0.8	*P* = 0.378
	Concentration (C)	2	0.00026	0.00013	2	1.3	*P* = 0.277
	Time (T)	4	0.00352	0.00088	30	8.9	***P* < 0.001**
	S × C	2	0.00013	0.00007	1	0.7	*P* = 0.520
	S × T	4	0.00053	0.00013	5	1.3	*P* = 0.263
	C × C	8	0.00092	0.00012	8	1.2	*P* = 0.332
	S × C × T	8	0.00035	0.00004	3	0.4	*P* = 0.890

*The effects in bold are significant at P < 0.05*.

### PLFA analysis

The obtained results revealed that vancomycin and/or *C*. *freundii* affected the structure of the soil microbial community during the 90-day experiment. The highest value for total PLFA biomass (347.42 nmol PLFA/g soil) was determined on day 1 for soil treated with vancomycin at a higher concentration (10 mg/kg soil) and inoculated with *C*. *freundii* (VA10+Cit), while the lowest value (212.90 nmol PLFA/g soil) was found in the control soil (C) on day 60 (Table 5). The three-way ANOVA analysis showed that the total PLFA biomass was significantly affected by the incubation time (*P* < 0.001) and the interactions between three tested factors, i.e., bacterial strain (S), vancomycin concentration (C), and time (T) (*P* < 0.001), which explained 21 and 19% of the variance, respectively (Table 5).

**Table 5:** **The total, bacterial and fungal biomass expressed as the biomass of phospholipid fatty acid marker (nmol PLFA/g soil) in vancomycin- and/or ***Citrobacter freundii***-treated soil**.

**Day**	**Treatment**	**Total PLFA**	**Bacterial PLFA**	**Bacteria**		**GP:GN**	**Fungi**	**Bacteria:fungi**
				**Gram-positive (GP)**	**Gram-negative (GN)**			
1	C	278.86 ± 47.31^b-h^	103.57 ± 11.52^b-f^	54.04 ± 4.54^d-h^	37.69 ± 6.67^b-e^	1.45 ± 0.13^def^	37.60 ± 13.12^a-e^	2.90 ± 0.62^jk^
	VA1	315.76 ± 13.22^ab^	120.22 ± 7.91^abc^	66.13 ± 6.38^abc^	41.88 ± 0.59^ab^	1.58 ± 0.13^a-d^	40.37 ± 5.09^ab^	3.02 ± 0.51^h-k^
	VA10	258.09 ± 59.53^b-i^	94.92 ± 19.06^def^	52.53 ± 9.48^e-i^	33.12 ± 7.08^cde^	1.59 ± 0.05^a-d^	35.79 ± 12.04^a-g^	2.77 ± 0.65^jk^
	Cit	259.76 ± 41.77^b-i^	94.06 ± 11.86^def^	50.20 ± 5.51^f-i^	32.87 ± 5.77^cde^	1.54 ± 0.11^bcd^	35.25 ± 8.01^a-g^	2.71 ± 0.35^jk^
	VA1+Cit	314.47 ± 44.85^abc^	121.06 ± 9.79^ab^	67.64 ± 3.32^ab^	40.94 ± 5.90^abc^	1.67 ± 0.15^ab^	37.74 ± 11.00^a-d^	3.33 ± 0.61^e-j^
	VA10+Cit	347.42 ± 38.41^a^	132.92 ± 12.85^a^	73.50 ± 7.71^a^	46.97 ± 4.65^a^	1.57 ± 0.12^b-d^	43.99 ± 9.32^a^	3.08 ± 0.49^h-k^
15	C	279.70 ± 45.83^b-h^	101.67 ± 17.85^b-f^	50.72 ± 8.56^f-i^	40.83 ± 7.76^abc^	1.25 ± 0.04^g^	31.17 ± 6.61^b-j^	3.29 ± 0.31^f-k^
	VA1	260.66 ± 40.12^b-i^	92.43 ± 15.54^def^	47.22 ± 7.91^f-i^	36.10 ± 6.35^b-e^	1.31 ± 0.02^fg^	33.95 ± 4.20^b-i^	2.71 ± 0.17^j-k^
	VA10	278.46 ± 43.47^b-h^	98.09 ± 16.74^c-f^	50.81 ± 9.36^f-i^	37.29 ± 5.71^b-e^	1.36 ± 0.06^efg^	37.05 ± 5.64^a-f^	2.64 ± 0.13^k^
	Cit	233.66 ± 16.25^e-i^	81.96 ± 6.40^f^	40.87 ± 3.00^i^	32.21 ± 2.90^cde^	1.27 ± 0.04^g^	28.59 ± 2.45^d-k^	2.87 ± 0.04^jk^
	VA1+Cit	293.32 ± 15.46^a-d^	104.23 ± 5.71^b-f^	53.64 ± 3.52^e-h^	40.48 ± 1.79^abc^	1.32 ± 0.03^fg^	39.29 ± 1.09^abc^	2.65 ± 0.07^jk^
	VA10+Cit	292.74 ± 13.23^a-e^	106.76 ± 4.59^b-e^	55.31 ± 2.89^c-g^	40.64 ± 1.53^abc^	1.36 ± 0.03^efg^	38.06 ± 3.68^a-d^	2.82 ± 0.20^jk^
30	C	256.90 ± 38.80^b-i^	101.05 ± 10.92^b-f^	50.42 ± 3.25^f-i^	38.62 ± 6.84^a-d^	1.33 ± 0.17^fg^	30.06 ± 10.35^c-k^	3.61 ± 1.10^d-i^
	VA1	259.31 ± 22.33^b-i^	99.76 ± 9.96^b-f^	50.41 ± 7.29^f-i^	38.19 ± 2.71^b-e^	1.32 ± 0.13^fg^	34.05 ± 1.98^b-i^	2.93 ± 0.15^jk^
	VA10	235.77 ± 7.87^d-i^	91.28 ± 2.80^ef^	46.56 ± 3.93^f-i^	35.07 ± 0.91^b-e^	1.33 ± 0.14^fg^	30.85 ± 0.51^b-j^	2.96 ± 0.08^ijk^
	Cit	226.90 ± 14.66^ghi^	86.42 ± 4.31^ef^	43.44 ± 1.38^ghi^	32.29 ± 4.94^cde^	1.37 ± 0.24^efg^	27.78 ± 3.08^e-l^	3.13 ± 0.22^g-k^
	VA1+Cit	237.41 ± 38.87^d-i^	90.62 ± 13.62^ef^	44.58 ± 4.04^f-i^	36.28 ± 7.60^b-e^	1.25 ± 0.18^g^	31.70 ± 6.94^b-j^	2.89 ± 0.20^jk^
	VA10+Cit	257.56 ± 11.94^b-i^	99.16 ± 1.43^b-f^	49.05 ± 2.23^f-i^	39.76 ± 0.71^abc^	1.23 ± 0.08^g^	34.11 ± 1.44^a-h^	2.91 ± 0.08^jk^
60	C	212.90 ± 72.01^i^	82.47 ± 30.61^f^	43.00 ± 17.07^hi^	29.47 ± 10.21^e^	1.44 ± 0.14^def^	20.49 ± 7.08^k-l^	3.99 ± 0.18^bcd^
	VA1	250.43 ± 11.41^d-i^	100.84 ± 4.30^b-f^	55.99 ± 2.20^b-f^	34.20 ± 1.93^b-e^	1.64 ± 0.04^abc^	23.92 ± 1.46^jkl^	4.22 ± 0.08^bcd^
	VA10	288.03 ± 34.70^a-f^	114.42 ± 14.66^a-d^	65.90 ± 9.27^a-d^	37.98 ± 4.41^b-e^	1.73 ± 0.04^a^	28.83 ± 2.14^d-k^	3.96 ± 0.29^cde^
	Cit	284.73 ± 6.51^b-g^	114.11 ± 2.24^a-d^	63.03 ± 1.73^a-e^	38.77 ± 0.44^a-d^	1.63 ± 0.03^abc^	24.66 ± 2.73^h-l^	4.67 ± 0.57^bc^
	VA1+Cit	264.49 ± 4.09^b-i^	102.62 ± 1.53^b-f^	54.81 ± 1.12^c-h^	37.92 ± 0.47^b-e^	1.45 ± 0.03^def^	26.56 ± 0.47^g-l^	3.86 ± 0.04^def^
	VA10+Cit	229.22 ± 82.74^f-i^	88.28 ± 35.46^ef^	47.91 ± 18.78^f-i^	30.47 ± 12.18^de^	1.58 ± 0.03^a-d^	23.47 ± 9.61^jkl^	3.77 ± 0.06^d-g^
90	C	250.47 ± 8.09^d-i^	98.94 ± 5.17^b-f^	51.68 ± 2.79^e-i^	34.68 ± 1.96^b-e^	1.49 ± 0.01^cde^	23.25 ± 0.53^jkl^	4.26 ± 0.17^bcd^
	VA1	220.63 ± 10.51^h-i^	89.50 ± 3.75^ef^	48.75 ± 1.74^f-i^	30.56 ± 1.34^de^	1.60 ± 0.03^a-d^	18.56 ± 1.61^l^	4.84 ± 0.22^b^
	VA10	247.50 ± 41.77^d-i^	98.12 ± 19.12^c-f^	54.03 ± 10.54^d-h^	34.31 ± 6.58^b-e^	1.57 ± 0.03^a-d^	24.16 ± 4.35^i-l^	4.08 ± 0.67^cd^
	Cit	236.19 ± 18.70^d-i^	98.35 ± 9.82^c-f^	52.55 ± 5.47^e-i^	34.09 ± 3.29^b-e^	1.54 ± 0.03^bcd^	17.91 ± 1.26^l^	5.53 ± 0.93^a^
	VA1+Cit	254.98 ± 22.67^c-i^	97.51 ± 9.17^def^	52.80 ± 5.22^e-i^	34.40 ± 3.26^b-e^	1.53 ± 0.01^bcd^	25.27 ± 2.13^h-l^	3.86 ± 0.11^def^
	VA10+Cit	260.06 ± 46.79^b-i^	101.04 ± 18.25^b-f^	55.07 ± 9.47^c-g^	35.73 ± 6.67^b-e^	1.54 ± 0.03^bcd^	27.66 ± 4.76^f-l^	3.65 ± 0.04^d-h^

*C, control soil; VA1, soil treated with vancomycin—1 mg/kg soil; VA10, soil treated with vancomycin—10 mg/kg soil; Cit, soil inoculated with C. freundii; VA1+Cit, soil treated with vancomycin—1 mg/kg soil and inoculated with C. freundii; VA10+Cit, soil treated with vancomycin—10 mg/kg soil and inoculated with C. freundii. The data presented are the means and standard deviations of three replicates. The different letters (within each group) indicate significant differences (P < 0.05, LSD test), considering the effects of the antibiotic dosage, bacterial strain and time*.

Similar results were obtained for the bacterial PLFA biomass whose value ranged from 82.47 nmol PLFA/g soil for the control soil (C) on day 60–132.92 nmol PLFA/g soil for the treated soil (VA10+Cit) on day 1 (Table 5). The bacterial PLFA biomass was shown to be significantly affected by the incubation time (*P* < 0.001) and the interactions between the three tested factors (S × C × T; *P* < 0.001). The interactions between the factors explained 24% of the variance, whereas the effect of the incubation time accounted for 13% of the variance (Table 6).

**Table 6:** **Results of three-way ANOVA for the effects of concentration of vancomycin, bacterial strain, time, and their interaction on the measured biomass of PLFA markers**.

**Parameter**	**Source of variation**	***df***	**Sum of squares**	**Mean square**	**Variance explained (%)**	***F***	***P***
TB	Strain (S)	1	988.88	988.88	1	0.7	*P* = 0.391
	Concentration (C)	2	5401.24	2700.62	3	2.0	*P* = 0.140
	Time (T)	4	33546.74	8386.68	21	6.3	***P* < 0.001**
	S × C	2	2319.90	1159.95	1	0.8	*P* = 0.422
	S × T	4	2740.26	685.07	2	0.5	*P* = 0.724
	C × T	8	5250.97	656.37	3	0.4	*P* = 0.855
	S × C × T	8	29874.42	3734.30	19	2.8	***P* = 0.010**
BB	Strain (S)	1	101.20	101.20	<1	0.5	*P* = 0.468
	Concentration (C)	2	708.66	354.33	3	1.8	*P* = 0.163
	Time (T)	4	2977.68	744.42	13	3.9	***P* < 0.010**
	S × C	2	295.20	147.60	1	0.7	*P* = 0.463
	S × T	4	535.12	133.78	2	0.7	*P* = 0.591
	C × T	8	1313.95	164.24	6	0.8	*P* = 0.549
	S × C × T	8	5455.48	681.94	24	3.6	***P* < 0.005**
GPBB	Strain (S)	1	26.30	26.30	<1	0.4	*P* = 0.487
	Concentration (C)	2	441.14	220.57	5	4.0	***P* < 0.050**
	Time (T)	4	1895.31	473.83	23	8.8	***P* < 0.001**
	S × C	2	17.53	8.77	<1	0.1	*P* = 0.850
	S × T	4	219.66	54.91	3	1.0	*P* = 0.404
	C × T	8	516.92	64.62	6	1.2	*P* = 0.314
	S × C × T	8	1909.74	238.72	23	4.4	***P* < 0.001**
GNBB	Strain (S)	1	19.04	19.04	<1	0.6	*P* = 0.418
	Concentration (C)	2	76.91	38.45	3	1.3	*P* = 0.269
	Time (T)	4	309.68	77.42	10	2.7	***P* < 0.050**
	S × C	2	117.37	58.69	4	2.0	*P* = 0.138
	S × T	4	46.34	11.58	2	0.4	*P* = 0.805
	C × T	8	115.73	14.47	4	0.5	*P* = 0.848
	S × C × T	8	657.61	82.20	21	2.8	***P* < 0.010**
GP:GN	Strain (S)	1	0.00	0.00	<1	0.1	*P* = 0.700
	Concentration (C)	2	0.05	0.03	2	2.6	*P* = 0.076
	Time (T)	4	1.41	0.35	60	36.9	***P* < 0.001**
	S × C	2	0.09	0.04	4	4.5	***P* < 0.050**
	S × T	4	0.03	0.01	1	0.8	*P* = 0.515
	C × T	8	0.11	0.01	5	1.4	*P* = 0.189
	S × C × T	8	0.08	0.01	4	1.0	*P* = 0.381
FB	Strain (S)	1	14.26	14.26	<1	0.3	*P* = 0.537
	Concentration (C)	2	358.49	179.25	6	4.8	***P* < 0.050**
	Time (T)	4	3151.01	787.75	51	21.3	***P* < 0.001**
	S × C	2	68.68	34.34	1	0.9	*P* = 0.400
	S × T	4	11.86	2.96	<1	0.1	*P* = 0.988
	C × T	8	81.68	10.21	1	0.2	*P* = 0.971
	S × C × T	8	319.27	39.91	5	1.1	*P* = 0.388
BB:FB	Strain (S)	1	0.02	0.02	<1	0.1	*P* = 0.730
	Concentration (C)	2	2.83	1.42	5	8.1	***P* < 0.001**
	Time (T)	4	36.00	9.00	62	51.9	***P* < 0.001**
	S × C	2	0.59	0.29	1	1.7	*P* = 0.191
	S × T	4	0.31	0.08	<1	0.4	*P* = 0.777
	C × T	8	2.80	0.35	5	2.0	*P* = 0.059
	S × C × T	8	5.19	0.65	9	3.7	***P* < 0.005**

*TB, total biomass; BB, bacterial biomass; GNBB, Gram-negative bacterial biomass; GPBB, Gram-positive bacterial biomass; FB, fungal biomass. The effects in bold are significant at P < 0.05*.

The biomass of the Gram-positive bacteria biomarkers was generally higher than those characteristic for Gram-negative bacteria with maximum values of 73.50 and 43.44 nmol PLFA/g soil, respectively (Table 5). The lowest value for the Gram-positive bacteria biomarker biomass (40.87 nmol PLFA/g soil) was determined for the soil inoculated with *C*. *freundii* (Cit) on day 14, while the lowest biomass for Gram-negative biomarkers (29.47 nmol PLFA/g soil) was observed for the control soil (C) on day 60 (Table 5). In both cases, the effect of the time as well as the interactions between the three tested factors (S × C × T) proved to be the major sources of the variance (10–23%). However, the effect of the concentration explained a further 5% of the variance for the biomass of Gram-positive bacteria (Table 6).

The differences in the abundance of the Gram-positive (GP) and Gram-negative (GN) bacteria biomarkers were also reflected by the GP:GN biomarker ratio (Table 5), which was primarily affected by the time factor, which explained most of the variance (60%). However, this parameter was also significantly (*P* < 0.05) affected by the interaction between the bacterial strain and vancomycin concentration (S × C), which explained only 4% of the variance (Table 6).

The fungal biomass was shown to be significantly affected by the incubation time (*P* < 0.001), which explained most of the variance (51%) as well as the concentration of vancomycin (*P* < 0.05); however, this factor explained only 6% of the variance (Table 6). The ratio of bacteria to fungi (BB:FB) also revealed a time-dependent variance (62%). The concentration of vancomycin and the interactions between the three tested factors (S × C × T) significantly affected (*P* < 0.001 and *P* < 0.005) the BB:FB ratio, which only explained 5 and 9% of the variance, respectively (Table 6).

**Figure 5:**
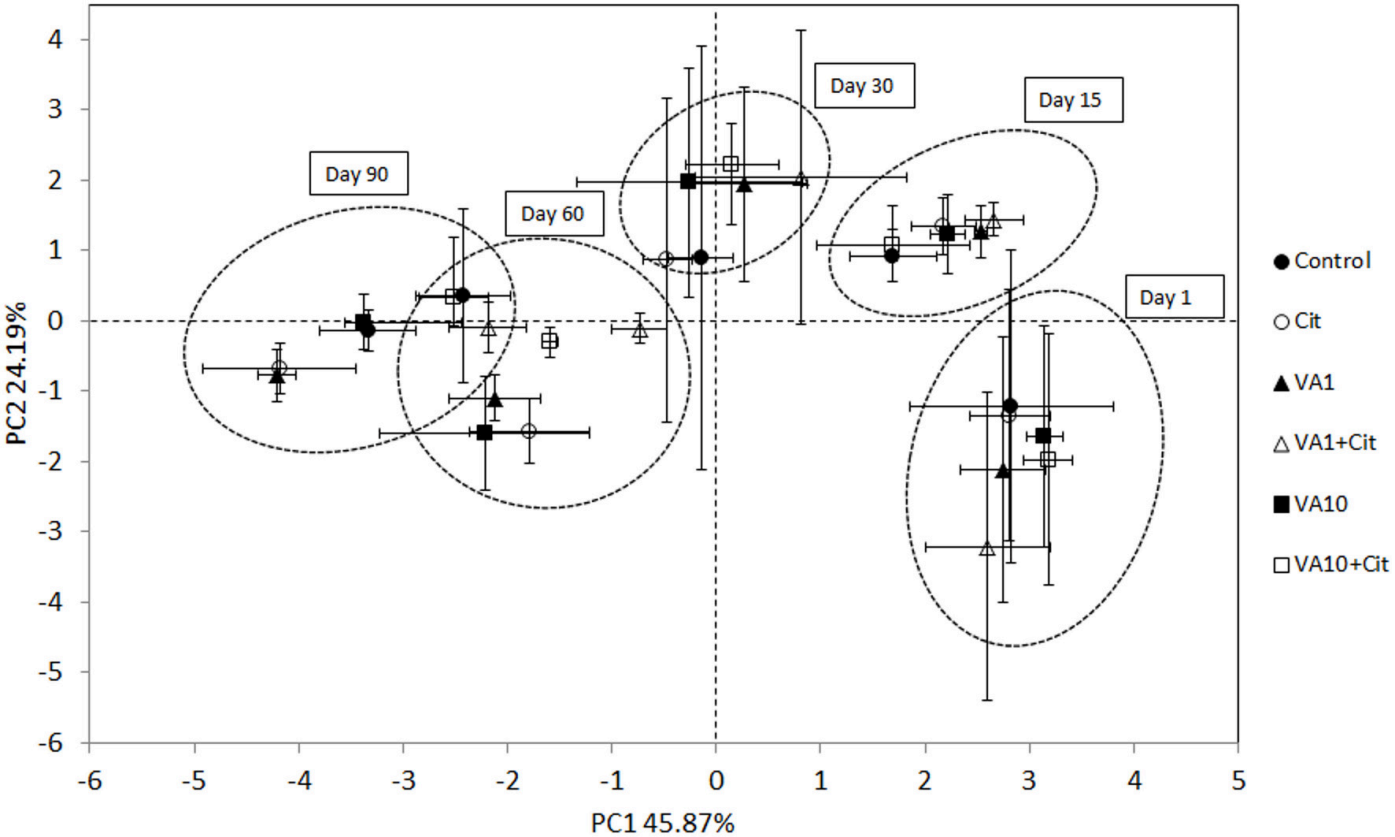
**Principal component plots generated from the standardized PLFA biomarker profiles on days 1, 15, 30, 60 and 90**. C, control soil; VA1, soil treated with vancomycin—1 mg/kg soil; VA10, soil treated with vancomycin—10 mg/kg soil, Cit, soil inoculated with *C. freundii*; VA1+Cit, soil treated with vancomycin—1 mg/kg soil and inoculated with *C. freundii*; VA10+Cit, soil treated with vancomycin—10 mg/kg soil and inoculated with *C. freundii*.

The PCA plot obtained for the standardized microbial biomarker profiles revealed a strong time-dependent pattern of variability. Distinct clusters of the samples that had been obtained on different sampling days were scattered along the PC1 axis, which explained 46% of the total variance, in contrast to the PC2 axis, which explained only 24% (Figure 5). This observation was confirmed by a three-way MANOVA analysis based on the scores from PC1 and PC2. The time contributed to 92 and 54% of the total variance observed along PC1 and PC2, respectively. Other factors, such as the concentration of vancomycin, bacterial strain as well as the interactions between three tested factors (S × C × T) explained <1% of the total variation observed in the PCA scatterplot (Table 7).

**Table 7:** **Result of three-way MANOVA for the PC1 and PC2 based on the data for all sampling days**.

**PC scores**	**Source of variation**	***df***	**Sum of squares**	**Mean squares**	**Variance explained (%)**	***F***	***P***
PC1	Strain (S)	1	2.79	2.79	<1	8.9	***P* < 0.050**
	Concentration (C)	2	4.15	2.08	<1	6.7	***P* < 0.050**
	Time (T)	4	489.69	122.42	92	395.0	***P* < 0.001**
	S × C	2	2.47	1.24	<1	3.9	***P* < 0.050**
	S × T	4	3.00	0.75	<1	2.4	*P* = 0.058
	C × T	8	4.11	0.51	<1	1.7	*P* = 0.127
	S × C × T	8	5.86	0.73	1	2.4	***P* < 0.050**
PC2	Strain (S)	1	0.00	0.00	<1	0.0	*P* = 0.989
	Concentration (C)	2	0.76	0.38	<1	0.2	*P* = 0.797
	Time (T)	4	152.52	38.13	54	22.7	***P* < 0.001**
	S × C	2	2.28	1.14	<1	0.6	*P* = 0.512
	S × T	4	1.51	0.38	<1	0.2	*P* = 0.924
	C × T	8	12.47	1.56	4	0.9	*P* = 0.501
	S × C × T	8	9.42	1.18	3	0.7	*P* = 0.690

*The effects in bold are significant at P < 0.05*.

**Figure 6:**
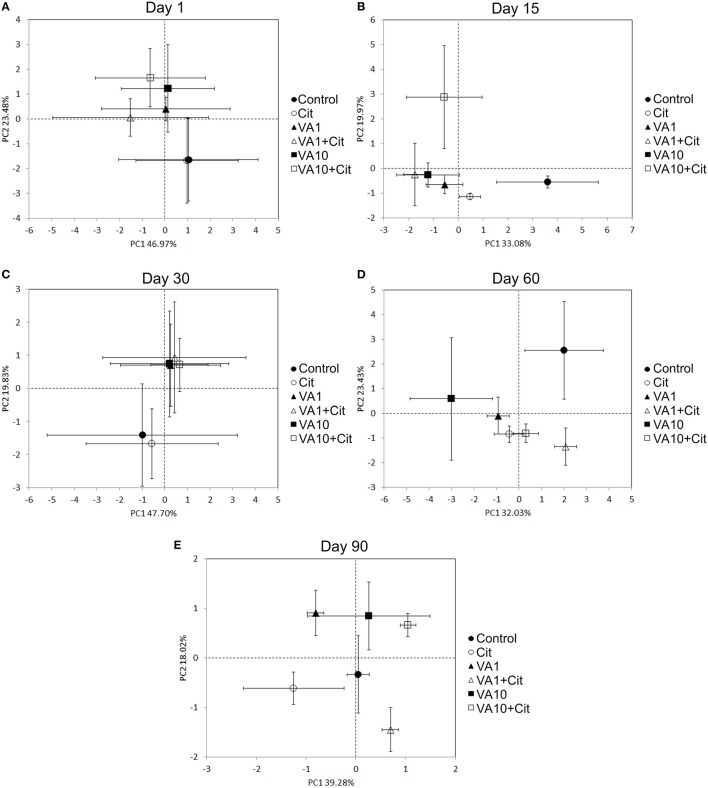
**Principal component plots generated from the standardized PLFA biomarker profiles on days 1 (A), 15 (B), 30 (C), 60 (D) and 90 (E)**. VA1, soil treated with vancomycin—1 mg/kg soil; VA10, soil treated with vancomycin—10 mg/kg soil; Cit, soil inoculated with *C*. *freundii*; VA1+Cit, soil treated with vancomycin—1 mg/kg soil and inoculated with *C*. *freundii*; VA10+Cit, soil treated with vancomycin—10 mg/kg soil and inoculated with *C*. *freundii*.

The PCA plots performed separately for individual sampling days enabled the impact of factors other than time of the observed variation within the microbial biomarkers to be investigated (Figures 6A–E). In general, a significant impact of the antibiotic concentration was evident up to day 30 of the experiment. This factor contributed to 57, 36, and 49% of the variance observed along PC2 on days 1, 15, and 30, respectively (Table 8). In addition, the effect of the interaction between the bacterial strain and the vancomycin concentration (S × C) was found along PC2 on day 15 and it explained 25% of the variance. In turn, a significant effect (*P* < 0.001) of the vancomycin concentration along PC1 was only observed on day 15, where this factor explained 51% of the variance (Figure 6B, Table 8). On days 60 and 90 of the experiment, in addition to the effect of the antibiotic concentration, the effect of the bacterial strain on PLFA profiles was also observed. This effect was mainly associated with PC2 and explained 35 and 23% of the variance on days 60 and 90, respectively (Table 8). In turn, the effect of the bacterial strain along PC1 was only observed on day 60 and explained 11% of the variance. Moreover, the interaction between the strain and the antibiotic concentration (S × C) was observed along PC1 and explained 44 and 37% of the variance on days 60 and 90, respectively (Table 8).

**Table 8:** **Results of two-way MANOVA for the PC1 and PC2 based on the data for each sampling day**.

**PC scores**	**Day**	**Source of variation**	***df***	**Sum of squares**	**Mean squares**	**Variance explained (%)**	***F***	***P***
PC1	1	Strain (S)	1	2.88	2.88	3	0.4	*P* = 0.546
		Concentration (C)	2	9.85	4.93	9	0.7	*P* = 0.534
		S × C	2	1.69	0.84	2	0.1	*P* = 0.894
	15	Strain (S)	1	6.76	6.76	9	4.5	*P* = 0.056
		Concentration (C)	2	37.50	18.75	51	12.4	***P* = 0.001**
		S × C	2	10.73	5.36	15	3.6	*P* = 0.061
	30	Strain (S)	1	0.55	0.55	<1	0.1	*P* = 0.802
		Concentration (C)	2	5.41	2.70	5	0.3	*P* = 0.728
		S × C	2	0.07	0.03	<1	0.0	*P* = 0.996
	60	Strain (S)	1	7.52	7.52	11	5.9	***P* < 0.050**
		Concentration (C)	2	16.59	8.30	23	6.6	***P* < 0.050**
		S × C	2	31.49	15.74	44	12.4	***P* = 0.001**
	90	Strain (S)	1	0.49	0.49	3	1.1	*P* = 0.314
		Concentration (C)	2	4.77	2.38	28	5.3	***P* < 0.050**
		S × C	2	6.37	3.19	37	7.1	***P* < 0.010**
PC2	1	Strain (S)	1	0.00	0.00	0.00	0.0	*P* = 0.980
		Concentration (C)	2	29.43	14.72	57	8.0	***P* < 0.010**
		S × C	2	0.45	0.23	1	0.1	*P* = 0.885
	15	Strain (S)	1	4.38	4.38	10	4.1	*P* = 0.066
		Concentration (C)	2	15.73	7.86	36	7.4	***P* < 0.010**
		S × C	2	11.23	5.61	25	5.3	***P* < 0.050**
	30	Strain (S)	1	0.00	0.00	<1	0.0	*P* = 0.971
		Concentration (C)	2	21.46	10.73	49	5.8	***P* < 0.050**
		S × C	2	0.19	0.09	<1	0.1	*P* = 0.950
	60	Strain (S)	1	18.23	18.23	35	10.1	***P* < 0.010**
		Concentration (C)	2	7.54	3.77	15	2.1	*P* = 0.167
		S × C	2	4.28	2.14	8	1.2	*P* = 0.340
	90	Strain (S)	1	3.97	3.97	23	14.4	***P* < 0.010**
		Concentration (C)	2	5.23	2.61	31	9.5	***P* < 0.010**
		S × C	2	4.51	2.25	27	8.2	***P* < 0.010**

*The effects in bold are significant at P < 0.05*.

## Discussion

Using an isolation procedure, a bacterial strain identified as *C. freundii* was screened. Citrobacter species, including *C. freundii*, are aerobic gram-negative bacilli. They can inhabit the environment (soils and water), food and the intestinal tracts of animals and humans (Wang et al., 2000). Since it is an opportunistic pathogen, *C. freundii* is often the cause of significant opportunistic infections, meaning that it does not generally cause diseases in healthy human hosts. *C. freundii* only affects patients with a weak immune system, thus signifying that they need an “opportunity” to infect a person (Whalen et al., 2007). Therefore, Citrobacter species are known to cause a wide variety of nosocomial infections of the respiratory tract, urinary tract and the blood in patients with a suppressed immune system. *C. freundii* represents ~29% of all opportunistic infections (Whalen et al., 2007). *C. freundii* has recently been reported to express resistance to broad-spectrum antibiotics including piperacillin, piperacillin-tazobactam, vancomycin, and cephalosporins (Kim et al., 2003). In our study, the disc diffusion and *E*-test methods showed that the isolated *C. freundii* was characterized by a resistance not only to vancomycin but also to clindamycin and erythromycin, for which the MICs exceeded 256 mg/mL. The entry of vancomycin-resistant bacteria into soil via the application of manure and sewage sludge produces a potentially significant reservoir of vancomycin-resistance genes (D'Costa et al., 2006; Gullberg et al., 2011). Once antibiotic-resistant bacteria and their corresponding suites of resistance genes enter the soil, the persistence and fate of these genes depend on the host bacteria that harbor the determinant(s) as well as the partitioning of free genetic material release from cells that may be uptake by new bacterial cells (Salyers and Amábile-Cuevas, 1997; Chee-Sanford et al., 2001).

The introduction of antibiotics and antibiotic-resistant microorganisms into soil leads to changes within the communities of soil microorganisms and the spread of antibiotic-resistance genes and consequently, to a reduction in the effectiveness of antibiotic therapy (D'Costa et al., 2006; Gullberg et al., 2011). It has been demonstrated that antibiotics affect the soil microbial processes, the microbial biomass, the respiration, and the microbial catabolic diversity after the application of these compounds to soil (Conkle and White, 2012; Rosendahl et al., 2012; Cui et al., 2013). For example, a significant decrease in soil activity and respiration as a response to sulfonamides, trimethoprim, and sulfadiazine exposure was reported by Liu et al. (2009) and Kotzerke et al. (2011). Sulfadiazine contamination also resulted in reduced denitrification rates in soil; however, this effect was only observed for a high antibiotic concentration (100 mg/kg soil). Schmitt et al. (2005), using the pollution-induced community tolerance (PICT) concept, found that a bacterial community extracted from soil contaminated with sulfachloropyridazine exhibited an increased tolerance to this antibiotic. Moreover, the analysis of community-level physiological profiles (CLPP), which was assessed using Biolog ECP plates, revealed that sulfachloropyridazine caused a statistically important shift in the metabolic activity of soil microbial communities as compared to those in the control soil. A decrease in functional diversity, evenness, average well color development, and substrate utilization of the bacterial populations in soil contaminated with oxytetracycline at concentrations higher than 43 μM (to 217 μM) was also revealed by Kong et al. (2006).

The results obtained in this study showed that vancomycin may contribute to qualitative and quantitative changes within indigenous microbial communities. The DGGE profiles of the vancomycin-treated samples differed from the control with the disappearance of some bands following antibiotic application. Changes in the bacterial communities of river sediments exposed to vancomycin at a concentration of 1000 μg/L were also reported by Laverman et al. (2015). Some previously described genotypic analyses of 16S rRNA gene fragment patterns on DGGE also indicated structural shifts within microbial communities through the loss or appearance of bands after various antibiotics were applied to soil (Zielezny et al., 2006; Hammesfahr et al., 2011). Changes in the DGGE banding profiles of 16S rRNA genes isolated from sediments contaminated with tetracycline were observed by Roose-Amsaleg et al. (2013). They found that both therapeutic (10,000 μg/L) and environmentally relevant tetracycline concentrations modified the bacteria composition as compared to non-exposed sediments. A modification of the composition of a bacterial community in anaerobic sediments as a response to ciprofloxacin (20 mg/mL) exposure was reported by Córdova-Kreylos and Scow (2007). Moreover, Fernandes et al. (2015) observed changes in the richness and diversity (assessed using ARISA) of microbial communities in a salt marsh treated with enrofloxacin. In addition, it has been shown that several clinically used antibiotics present in natural environments can select the antibiotics-resistant bacteria (Gullberg et al., 2011; Tang et al., 2015).

The results of our study and other studies may be proof that species sensitive to vancomycin or other antibiotics were killed or their number decreased substantially. The consequence of these changes may be an increase in the number of specific vancomycin-tolerant/resistant bacteria and a decrease in the overall richness (S) and the diversity (H) of the members of bacterial communities. As was revealed by Iweriebor et al. (2015), vancomycin-resistant bacteria have the ability to transfer the vancomycin-resistance determinant to other bacteria and this poses a serious threat to human health. A number of studies have recently shown that the transfer of antibiotic-resistance genes between bacteria occurs frequently in natural ecosystems and hospitals (Onan and LaPara, 2003; Riesenfeld et al., 2004). It has been revealed that the plasmid carrying the Tn1546-like was the main factor in the spread of vancomycin-resistance genes (VanA gene cluster) in enterococcal populations that contributed to an outbreak in hospital (Kawalec et al., 2000).

The changes in the composition of a microbial community based on PLFA analysis observed in this study were also observed by Hund-Rinke et al. (2004) for soils that had been spiked with tetracycline. The authors found a decrease in the amount of specific PLFA biomarkers, which was accompanied by a reduction in the growth of Gram-positive bacteria. The results of a study carried out by Reichel et al. (2013) confirmed the influence of slurry containing sulfadiazine and difloxacin at field-relevant concentrations on the structural diversity of soil microbial communities. They observed a temporal shift and dominance of Gram-negative bacteria in soils that had been treated with both antibiotics as indicated by a decreased value of the Gram-positive to Gram-negative ratios. Moreover, they occasionally found a shift toward a higher fungal to bacterial biomass content as reflected by decreased bacteria:fungi PLFA ratios in the antibiotic-treated soils. The reduced bacterial competitiveness, which was often followed by the intensive growth of fungi in antibiotic-polluted soil, was observed by many authors (Hund-Rinke et al., 2004; Hammesfahr et al., 2008; Demoling et al., 2009; Gutiérrez et al., 2010). Reichel et al. (2013) showed that changes in the total PLFA biomass and PLFA patterns obtained from soils treated with sulfadiazine-contaminated manure not only depended on antibiotic application but also on the soil microhabitat (bulk soil, rhizosphere, earthworm burrows, interiors, and surface of aggregates). They stated that the properties of a microhabitat dominated the structure of microbial composition and were reflected by the different responses of communities toward sulfadiazine.

In conclusion, the results of this study indicated that the pollution of soil by vancomycin and/or vancomycin-resistant bacteria negatively affected the structure of microbial communities. DGGE analysis confirmed that certain species within the bacterial community were sensitive to vancomycin as was evidenced by the absence of some DGGE bands and decrease in the values of biodiversity indices. However, it can be assumed that the number of species characterized by a higher tolerance/resistance to this antibiotic will increase over time. The observed changes in the structure of soil bacteria may also result in a lower rate of important soil processes, which can lead to the disturbance of the ecological balance of the soil ecosystem as well as in the spread of vancomycin-resistance genes.

## Author contributions

Conceived and designed experiments: MC. Contributed reagents and materials, performed experiments: MC, SB, and KO. Analyzed results: MC, SB, TW, and ZP. Wrote the paper: MC, SB, and ZP.

### Conflict of interest statement

The authors declare that the research was conducted in the absence of any commercial or financial relationships that could be construed as a potential conflict of interest.
